# Cranial Nerve Impairment Associated With COVID-19 Infections: A Systematic Review

**DOI:** 10.7759/cureus.31997

**Published:** 2022-11-28

**Authors:** Albaraa Tonkal, Abdullah A Alamri, Sahar J AlMaghrabi, Naif F Mozahim, Sarah F Mozahim, Shahad A Alsubaie, Areej A Alsehly, Razan O Alshuaibi, Leena A Alotaibi, Fadi S Qashgari

**Affiliations:** 1 Otolaryngology - Head and Neck Surgery, King Abdulaziz University Hospital, Jeddah, SAU; 2 Otolaryngology - Head and Neck Surgery, King Abdulaziz University, Jeddah, SAU; 3 Otolaryngology - Head and Neck Surgery, King Abdulaziz University Faculty of Medicine, Jeddah, SAU; 4 Medical Microbiology, College of Medicine, Umm Al Qura University, Makkah, SAU

**Keywords:** covid-19 neurological outcomes, neurological signs and symptoms, cns involvement, cranial nerve palsies, covid-19

## Abstract

The COVID-19 pandemic has created huge economic and healthcare burdens. In most cases, the virus affects the lungs and causes respiratory symptoms. Additionally, its impact on the cranial nerves remains unclear. We thus aimed to investigate cranial nerve dysfunction in patients with COVID-19 infection.

We conducted a systematic literature search of relevant and eligible literature in five databases: PubMed, Web of Science, Medline, EBSCO, and Google Scholar.

Our sample included 21 case reports, one case series with 29 patients, and one analytical study with 135 cases. Participant ages ranged from 23 months to 72 years (mean age of 47.5 ± 19.02). The mean time from respiratory symptoms to the onset of neurological signs was (9.6 ± 7.4) days, and the mean recovery time was (16.3 ± 15.3) days.

Cranial nerve impairment associated with COVID-19 infection has affected a large population, from infants to the elderly. Facial and abducent nerves were the most commonly affected cranial nerves with reported good prognosis or complete recovery within a few days to weeks. Olfactory dysfunctions were widely detected among COVID-19 patients.

## Introduction and background

Coronaviruses are typically considered respiratory pathogens. However, neurologic complications such as confusion, stroke, seizure, and neuromuscular disorders have been associated with these viruses, particularly in those with severe infections [1-4].

In 2002, an outbreak of SARS-CoV-1, a member of the coronavirus family of viruses, induced a series of neurological disorders, including encephalopathy, stroke, seizures, cranial nerve dysfunction, peripheral neuropathy, and myopathy. The death rate of around 10% helped limit the spread of the disease [5,6]. However, in 2012, another coronavirus, Middle East Respiratory Syndrome coronavirus (MERS), spread across the Middle East [5]. MERS causes multiple organ disorders affecting the brain, nerves, and muscles [7]. 

In 2019, the COVID-19 pandemic was caused by a coronavirus with high SARS-CoV-1 and MERS homology that affects both the central and peripheral nervous systems [4,8,9]. COVID-19 caused a global health and economic crisis, and around 50 million people worldwide have been infected [10,11]. 

The pathophysiology of nerve injury is neuronal swelling and edema of the brain inducing neurological damage, peripheral vasodilatation, hypercarbia, hypoxia, and anaerobic metabolism [12]. An investigation in China has reported a higher incidence of neurological symptoms in severe cases of COVID-19 [3]. Further investigation is needed to detect the impact of the COVID-19 virus on neurological manifestations, particularly cranial nerve involvement such as facial nerve palsy and loss of taste and smell. 

This systematic review, conducted between August and September 2021, aimed to summarize the published literature regarding COVID-19 patients with cranial nerve impairment.

Using five essential databases (PubMed, Web of Science, Medline, Google Scholar, and EBSCO), we conducted a systematic literature search. We limited our search to papers written in English and used keywords compliant with PubMed’s Medical Subject Headings (MeSH) terms, including "COVID-19," "SARS-CoV-2," "Coronavirus Disease-2019", "2019-novel coronavirus", "severe acute respiratory syndrome coronavirus 2", "Cranial nerve," "neurological manifestations," and "CNS." Keywords were combined with Boolean operators such as "OR" and "AND." 

We then selected studies that met the following selection criteria: case reports, case series, and analytical studies of COVID-19 associated with cranial nerve involvement or neurological manifestations involving patients of any age. We excluded papers not written in English language or with limited access (e.g., paywalls). We then used Rayyan for Systematic Reviews (Rayyan Systems Inc., Cambridge, USA) to identify and remove duplicate records [13]. 

After screening abstracts according to the inclusion and exclusion criteria, the whole texts of eligible publications were evaluated by the reviewers. Any disagreements were resolved via debate and discussion. A data extraction form was used to record information from the qualifying articles. This information included the study topic, authors, year, design, and population, as well as the number of participants, their ages, genders, diagnoses, medical histories, presentation at admission, and treatments. Neurological signs (cranial nerve involvement, number of days from early respiratory symptoms to neurological symptoms, and time to recovery) also were recorded.

We evaluated the quality of the included case reports and the risk of bias using Joanna Briggs Institute software (JBI, Adelaide, Australia), discussing any discrepancies that arose. We then organized all results in tables, including research characteristics and data outcomes. After completing the data extraction, a qualitative analysis of the collected data was conducted.

## Review

**Figure 1:**
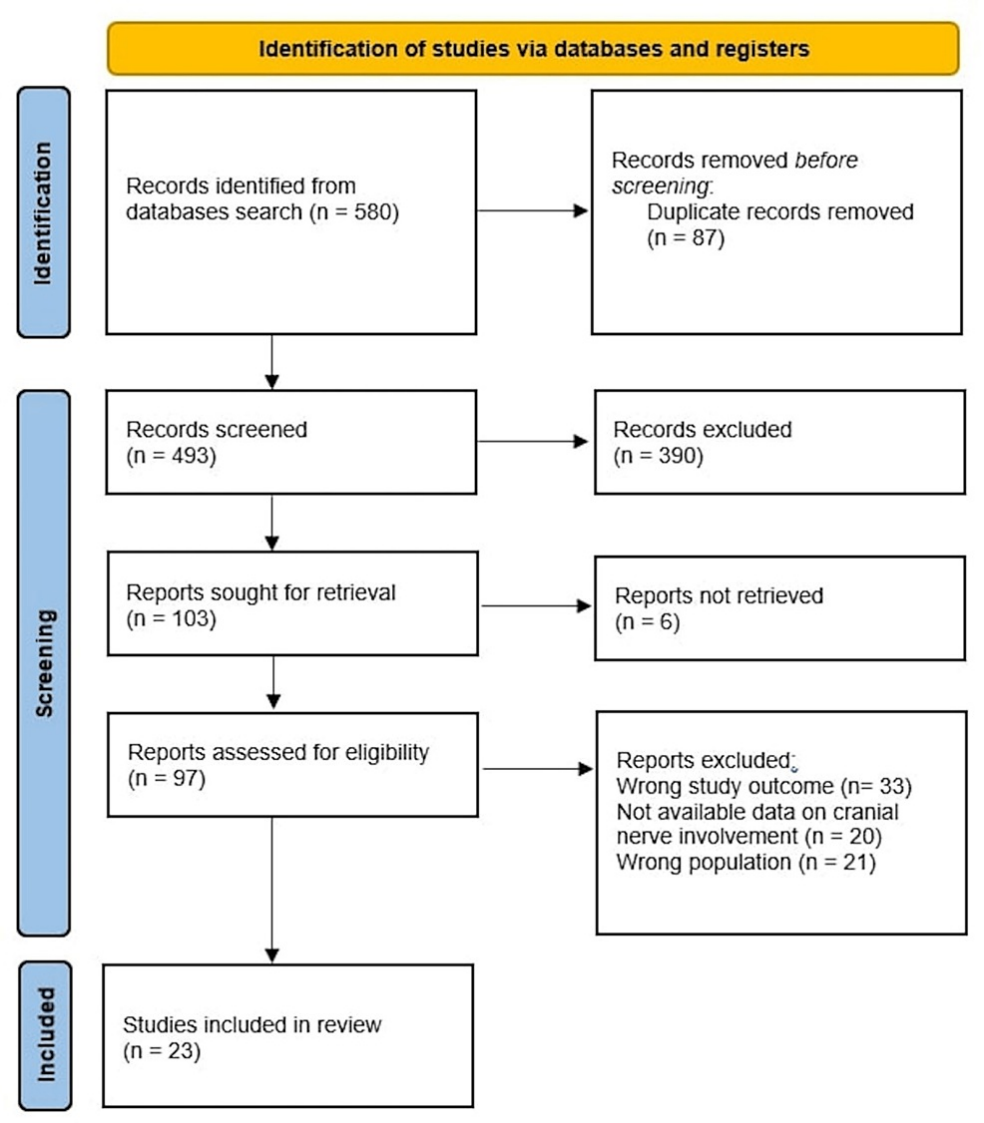
PRISMA flowchart presenting a summary of the study selection process. PRISMA: Preferred Reporting Items for Systematic Reviews and Meta-Analyses

The first systematic search yielded 580 studies, from which Rayyan identified 87 duplicate records. Another 390 were removed due to irrelevant findings or incorrect research type or design. The whole-text assessment step eliminated 75 more papers due to improper outcome, wrong population, or unavailable data on cranial nerve involvement. The final set thus comprised 23 eligible articles. Figure 1 illustrates the selection and identification process.

These 23 eligible articles included 21 case reports, one series comprising six cases, and one analytical study with 135 cases. Participant ages ranged from 23 months to 72 years, with a mean age of 47.5 ± 19.02. Six were done in the U.S, Three in Italy, two in Spain, seven in Brazil, two in India, two in France, one in England, one in Portugal, one in Japan, one in Bangladesh, one in Kuwait, one in Qatar, one in Turkey, and one in Morocco [14-36]. Most cases presented with general symptoms, such as hyperthermia, shortness of breath, cough, fatigue, anosmia, loss of sense of taste, nausea, vomiting, and diarrhea. 

The most frequently affected cranial nerves among COVID-19 patients were the facial nerve (26%) and the abducens nerve (12%). [15-18,22,23,25,26,28,29,34-36]. The mean time from respiratory symptoms to the onset of neurological signs was 9.6 ± 7.4 days, and the mean recovery time was 16.3 ± 15.3 days. The most frequent neurological manifestations in facial nerve paralysis were the inability to close one eye, drooping on one side of the mouth, loss of forehead wrinkling on the affected side, and deviation of angle of the mouth towards the opposite side along with drooling of saliva on the right side [19,23 ]. Regarding sixth-nerve palsy, diplopia was the most common sign [25,26,28,29,34].

Absence of gag reflex, less effective voluntary and reflex cough, oropharyngeal dysphagia, altered sense of taste, tongue deviation, and paralysis of vocal cords were the most common neurological manifestations in ninth- and tenth-nerve palsies. Ptosis, double vision, strabismus, and blurred vision were the most frequent neurological manifestations in second and third-nerve paralysis. Odynophagia was reported in hypoglossal nerve paralysis. Loss of sense of taste and smell was detected in olfactory nerve affection [16,18,20,21,24,30-34,37-38]. 

Generally, children were less symptomatic than adults, but neurological manifestations were observed in children with extrapulmonary symptoms. A study of 27 children with COVID-19 pediatric multisystem inflammatory syndrome (MIS-C) showed that 14.8% had acute onset of central nervous system (CNS)symptoms, including brain parenchyma causing encephalopathy, weakness, headaches, loss of reflexes, and cerebellar dysfunction [39]. 

Furthermore, one study reported a higher incidence of facial paralysis during the COVID-19 pandemic than in the same period in 2020, indicating a possible link between COVID-19 and peripheral facial nerve paralysis [40]. Facial nerve paralysis has also been associated with infections, most commonly herpes simplex virus, varicella-zoster, human immunodeficiency viruses, Lyme disease, and mycobacterium tuberculosis [41]. Also, vagus and hypoglossal nerve impairment were reported which can lead to swallowing difficulty [42]. However, the reported dysphagia was mostly associated with prolonged endotracheal intubation [43].

Olfactory nerve dysfunction leading to an impaired sense of smell and taste which was common among patients with COVID-19 infection and was seen to persist after the resolution of other symptoms in 63% of patients. [44]. However, most patients with olfactory dysfunctions experience the onset of olfactory impairment at the same time as COVID-19 infection [45]. Other studies have reported that taste dysfunction in COVID-19 occurs more often than olfactory impairment, and 10.2-22.5% of patients have impaired taste without olfactory dysfunction [44,46,47]. Table 1 summarizes the results.

**Table 1:** Summary of sociodemographic and clinical characteristics of the included studies. DM: diabetes mellitus; T2DM: type 2 diabetes mellitus; COPD: chronic obstructive pulmonary disease; JBI: Joanna Briggs Institute software

Study author	Study design	Country	Age (Years)	Sex	Presentation/ signs	Medical history	Neurological signs	Diagnosis in addition to Covid-19	Cranial nerve involved	Treatment	# days from respir-atory to neuro-logical symptoms	# days to recover	JBI
Doblan et al. 2021 [14]	Analytical (N=135)	Turkey	39.3 ± 16.4	Males: 71 (52.6%)	Fever (34.8%) Sore throat (32.6%) Cough (27.4%) Tiredness (25.9%) Headache (23.7%) Diarrhea (9.6%) Difficulty breathing (8.1%) Joint pain (10.4%) Hoarseness (2.2%)	Hypertension (9.6%) DM (5.9%) Cardiac disease (3.7%) Asthma/COPD (7.4%) Behcet’s (0.7%)		None	N. olfactorius (27.2%) N. opticus (5.0%) N. oculomotorius (4.0%) N. trochlearis (1.7%) N. trigeminus (1.7%) N. abducens (0.7%) N. facialis (30.8%) N. vestibulocochlearis (17.2%) N. glossofarengeus (25.2%) N. vagus (9.3%) N. accessories (3.6%) N. hypoglossus			3-23 in hospital	7
Gogia et al. 2020 [15]	Case report	USA	58	Male	Chest pain Nausea Vomiting Shortness of breath Abdominal pain Fever 5 days before admission	COPD Hypertension Non-obstructive coronary artery Facial trauma without permanent impairment	Left side facial numbness Dribbling across left side of face Mild dysphagia	Multiple cranial neuropathies	Trigeminal and facial	Valacyclovir (1 g) 3 times/day for 7 days Remdesivir for 5 days then convalescent plasma and dexamethasone	4	7	7
Kopscik et al. 2020 [16]	Case report	Spain	31	Male	None	None	Progressively worsening weakness Numbness Difficulty walking Double vision	Acute motor and sensory polyneuropathy	Abducent, facial, and hypoglossal	Physical/ occupational therapy Convalescent plasma Tocilizumab Intravenous immunoglobulin	7	NA	6
Cabrera et al. 2021 [17]	Case report	Spain	20	Male	Significant asthenia Headache Myalgia Nausea Vomiting	None	Acute right facial weakness	Co-infection of Epstein Barr virus w/bilateral facial nerve palsy	Facial	Levofloxacin 500 mg 1 x/day for 7 days Tapering with prednisone 60 mg/24 h	7	21	6
Kamel et al. 2019 [18]	Case report	Kuwait	55	Male	Fever Myalgia Persistent cough	Diabetes Hypertension Nonfunctioning pituitary macroadenoma	Severe headache Acute onset ptosis Diminution of vision in left eye Dilated nonreactive pupil	Pituitary apoplexy	Optic and oculomotor	Levothyroxine and hydrocortisone for panhypopituitarism	6	NA	7
Zain et al. 2021 [19]	Case report	USA	23 months	Female	None	None	Inability to fully close right eye Drooping of right side of mouth	Facial nerve neuritis	Facial	Dextrose 5 % in normal saline for hydration. Bell's palsy prompted 1 mg/kg/day of methylprednisolone 10- day steroid course with short taper for neurological symptoms	NA	21	6
Cavalagli et al. 2020 [20]	Case report	Italy	69	Male	Fever Dyspnea	Patent foramen ovale Heavy smoker Overweight Anamnesis Familial history of chronic anxiety	Global muscular hypotrophy Diminished patellar/Achilles' tendon reflexes on right side Tongue deviation and hypotrophy on right side Bilateral absence of gag reflex Ineffective voluntary and reflex cough	Cranial nerve impairments	Trigeminal, glossopharyngeal, vagus, and hypoglossal	Rehabilitative treatment	34	56	7
Fitzpatrick et al. 2021 [21]	Case report	USA	67	Male	None	Lyme disease	Double vision Left ptosis	3rd nerve palsy	Oculomotor	NA	4	NA	6
Vasanthapuram et al. 2021 [22]	Case report	India	58	Male	None	None	Vertical diplopia Enhanced downgaze and levoversion Left eye exotropia/ hypotropia 15° Limited adduction in right eye Left-beating nystagmus in left eye on abduction	Internuclear ophthalmoplegia	Oculomotor and abducent	Vitamin B12 supplements and ivermectin daily Oral doxycycline 2 x/day Vitamin C for 10 days Metformin 500 mg/day	21	30	6
Kumar et al. 2021 [23]	Case report	India	28	Female	Fever (1-day duration) Anosmia with dysgeusia	Polycystic ovarian disease	Loss of right forehead wrinkling Inability to close right eye (Bell's phenomenon) Deviation of angle of mouth towards left Drooling on right side	Lower motor neuron facial palsy	Facial	Oral valacyclovir 1 g 3 x/day for 10 days Oral prednisolone 50 mg/day for 7 days followed by rapid tapering	NA	NA	7
Aoyagi et al. 2020 [24]	Case report	Japan	70	Male	None	Prostate cancer Hypertension	Oropharyngeal dysphagia Altered sense of taste Absent gag reflex	Oropharyngeal dysphagia	Glossopharyngeal and vagus	Favipiravir 1600 mg 2 x/day Intravenous ampicillin sodium 2 g Sulbactam sodium 1 g/day for superimposed aspiration pneumonia	20	NA	7
Francis et al. 2021 [25]	Case report	France	69	Female	Anosmia	None	Acute, binocular, horizontal diplopia	Left abducens nerve palsy	Abducent	NA	8	NA	5
Srijon et al. 2020 [26]	Case report	Bangladesh	55	Female	Fever Cough	Hypertension Diabetes	Marked diplopia on right lateral gaze Right-sided convergent squint with restriction of right lateral gaze	Right abducens nerve palsy	Abducent	IV Methyl Prednisolone IV remdesivir Subcutaneous enoxaparin Supplemental oxygen Other symptomatic management	2	7	7
Oliveira et al. 2020 [27]	Case report	Brazil	69	Male	Fever (38°C) Abdominal pain Left posterior chest pain without cough or dyspnea Mild occipital headache	None	Binocular diplopia Severe stabbing occipital headache	Bilateral trochlear nerve palsy	Trochlear	IV methylprednisolone for 5 days with complete improvement of pain and diplopia	11	5	6
Anilkumar et al. 2021 [28]	Case report	England	44	Female	None	None	Persistent diplopia Mild right-side headache Blurred vision	6th nerve palsy	Abducent	Paracetamol for pyrexia	5	NA	6
Aldeeb et al. 2021 [29]	Case report	Qatar	48	Male	Vomiting Cough Diarrhea	None	Binocular diplopia more pronounced on looking to left Clear limitation of abduction in left eye with left gaze	6th nerve palsy	Abducent	Hydroxychloroquine Azithromycin Ceftriaxone Eye cover for diplopia	2	10	6
Belghmaidi et al. 2021 [30]	Case report	Morocco	24	Female	Fever (38.5°C) Dry cough Anosmia	None	Acute onset of diplopia Strabismus of left eye	3rd nerve palsy	Oculomotor	Chloroquine 500 mg 2 x/day for 10 days with azithromycin 500 mg/day the first day then 250 mg every day for 6 days) Vitamin C 1 g 2 x/day for 10 days Zinc 90 mg 2 x/day for 10 days	3	6	6
Decavel et al. 2020 [31]	Case report	France	62	Male	Fever Cough	Arterial hypertension and type II diabetes (T2DM)	Left hypoglossal nerve paralysis with tongue deviation towards left Complete paralysis of left vocal cord in abducted position	Left hypoglossal and vagus nerve paralysis	Glossopharyngeal Vagus	NA	16	30	6
Douedi et al. 2021 [32]	Case report	USA	55	Male	Generalized and bilateral headache graded 2-3/10 Generalized fatigue Loss of sense of taste Double blurry vision	Seizures disorder on levetiracetam	Left-side ptosis and diplopia on all fields of gaze except left	3rd nerve palsy	Oculomotor	NA	6	3	6
Costa Martins et al. 2020 [33]	Case report	Portugal	24	Male	Fever Respiratory distress with hypotension Tachycardia Tachypnea	None	Odynophagia Headache	Unilateral hypoglossal nerve palsy	Hypoglossal	Fentanyl, propofol, and rocuronium for rapid sequence intubation Biperiden for akinetic rigid syndrome Extubated on day 13 and started daily rehabilitation with physical therapy	2	43	6
Dinkin et al. 2020 [34]	Case report	USA	36	Male	Fever Cough Myalgia	Infantile strabismus	Left ptosis Diplopia Bilateral distal leg paresthesia Lower extremity hyporeflexia and hypesthesia Gait ataxia	Ophthal-moparesis	Abducent	Intravenous immunoglobulin 2g/kg for 3 days to manage presumed Miller Fisher Syndrome Hydroxychloroquine 600 mg 2 x/day for 1 day, followed by 400 mg/day for 4 days	14	NA	6
			71	Female	Cough Fever Hypoxia	Hypertension	Painless diplopia on waking two days prior Could not abduct right eye	Ophtha-lmoparesis	Optic Abducent	Hydroxychloroquine 600 mg 2 x/day for 1 day followed by 400 mg/day for 4 days	4	14 after discharge	
Manganotti et al. 2021 [35]	Case report	Italy	72	Male	Fever Dyspnea Hyposmia Ageusia	None	Flaccid tetraparesis with proximal upper limb predominance	Guillain‐Barré syndrome Polyneuritis cranialis	Facial	IVIG cycle 0.4 g/kg for 5 days for neurological symptoms Hydroxychloroquine Oseltamivir Darunavir Methylprednisolone Tocilizumab	6	NA	7
			49	Female	Fever Cough Dyspnea Hyposmia Ageusia	None	Ophthalmoplegia with diplopia in vertical and lateral gaze Limb ataxia	Guillain‐Barré syndrome Polyneuritis cranialis	Trigeminal Facial	IVIG cycle 0.4 g/kg for 5 days for neurological symptoms. Hydroxychloroquine Lopinavir-ritonavir Methylprednisolone	10	NA	
Corrêa et al. 2021 [36]	Case series	Brazil	41	Female	Malaise Cough	None	Loss of sense of taste and smell	Olfactory nerve affection	Olfactory	None	14	NA	6
			27	Female	Fever Cough	None	Blurred vision and pain in left eye Clinical suspicion of optic neuritis	Optic nerve affection	Optic	Anti-aquaporin-4 antibody was negative in serum Methylprednisolone 1 g/day for 5 days	14	5	
			25	Female	Mild dyspnea Fever	None	Vertigo Muscle weakness in right side of the face Difficulty closing right eye Strabismus in right eye	Abducent and facial nerve paralysis	Abducent Facial	Oral prednisone 60 mg/day	4	7	
			30	Female	Mild fever Sore throat	None	Right facial nerve palsy	Right facial nerve paralysis	Facial	Oral prednisone 60 mg/day	NA	5	
			65	Male	Osteoarthritis Atrial fibrillation	None	Left facial nerve palsy Lower limb weakness	Bilateral facial nerve paralysis	Facial	Intravenous immunoglobulin	18	NA	
			33	Male	Fever	None	NA	Bilateral facial nerve paralysis	Facial	oral prednisone 60 mg/day for 7 days	14	7	

## Conclusions

Our systematic review showed that the sixth and seventh cranial nerves were most affected among COVID-19 patients, and most symptoms involved isolated facial paralysis with mild to moderate impairment and no other neurological signs. Supportive care and oral steroids are the mainstays of reported treatment. Patients had complete recovery or noticeable improvement in a few days to weeks after starting the treatment, suggesting a favorable prognosis for peripheral facial palsy associated with COVID-19. Treatment for cases involving sixth-nerve palsy target management of COVID-19 and its complications. Also, olfactory nerve impairment with loss of smell and taste sensations was widely detected among COVID-19 patients.

Vagus and hypoglossal nerve impairment were reported in this review, along with the absence of the gag reflex, less effective voluntary and reflex cough, oropharyngeal dysphagia, altered sense of taste, tongue deviation, and paralysis of vocal cords. The vagus nerve and its branches supply multiple muscles in the head and neck in addition to their sensitive, sensory, and vegetative parts. 

Ophthalmological manifestations due to third-nerve palsy affecting the optic nerve also were reported in this review. Symptoms included ptosis, double vision, and blurred vision. Supportive treatment and eye care were the most effective management strategies. 

It is possible that some of the neurological manifestations reported in this review may not be associated with COVID-19 infection and are instead coincidental co-morbidities in the patient. Moreover, the associated sepsis and organ failure in patients with serious COVID-19 infection led to various neurological presentations that typically present in any critical condition. More research on neurological manifestations associated with COVID-19 infection is needed to determine if these observed symptoms are due to possible side effects from medication used to treat COVID-19.
